# Antagonism of human CC-chemokine receptor 4 can be achieved through three distinct binding sites on the receptor

**DOI:** 10.1002/prp2.19

**Published:** 2013-12-30

**Authors:** Robert J Slack, Linda J Russell, Nick P Barton, Cathryn Weston, Giovanna Nalesso, Sally-Anne Thompson, Morven Allen, Yu Hua Chen, Ashley Barnes, Simon T Hodgson, David A Hall

**Affiliations:** 1 Lead Optimisation, Respiratory CEDD, GlaxoSmithKlineGunnels Wood Road, Stevenage, Herts, SG1 2NY, UK; 2 Computational Chemistry, GlaxoSmithKlineGunnels Wood Road, Stevenage, Herts, SG1 2NY, UK; 3 Biological Reagents and Assay Development, GlaxoSmithKlineGunnels Wood Road, Stevenage, Herts, SG1 2NY, UK; 4 Medicinal Chemistry, Respiratory CEDD, GlaxoSmithKlineGunnels Wood Road, Stevenage, Herts, SG1 2NY, UK

**Keywords:** Allosteric modulator, CCL17, CCL22, CCR4, CCR4 antagonist, chemokine, T cell

## Abstract

Chemokine receptor antagonists appear to access two distinct binding sites on different members of this receptor family. One class of CCR4 antagonists has been suggested to bind to a site accessible from the cytoplasm while a second class did not bind to this site. In this report, we demonstrate that antagonists representing a variety of structural classes bind to two distinct allosteric sites on CCR4. The effects of pairs of low-molecular weight and/or chemokine CCR4 antagonists were evaluated on CCL17- and CCL22-induced responses of human CCR4^+^ T cells. This provided an initial grouping of the antagonists into sets which appeared to bind to distinct binding sites. Binding studies were then performed with radioligands from each set to confirm these groupings. Some novel receptor theory was developed to allow the interpretation of the effects of the antagonist combinations. The theory indicates that, generally, the concentration-ratio of a pair of competing allosteric modulators is maximally the sum of their individual effects while that of two modulators acting at different sites is likely to be greater than their sum. The low-molecular weight antagonists could be grouped into two sets on the basis of the functional and binding experiments. The antagonistic chemokines formed a third set whose behaviour was consistent with that of simple competitive antagonists. These studies indicate that there are two allosteric regulatory sites on CCR4.

## Introduction

The chemokines are a family of small (predominantly 8–10 kDa) proteins which act as leucocyte chemoattractants. They may be subdivided into four families based on the arrangement of the first two of four conserved cysteine residues. The largest of these families are the CC-chemokines in which the cysteine residues are adjacent and the CXC-chemokines in which the cysteines are separated by an intervening amino acid residue (Zlotnik and Yoshie 2000). The chemokine receptors are G_i_-protein coupled receptors and are also divided into four families, based on their ligand specificity, for example CC-chemokines are agonists for CC-chemokine receptors while CXC-chemokine receptors respond only to CXC-chemokines (Murphy et al. 2000).

CCR4 is the receptor for the CC-chemokines CC-chemokine ligand (CCL) 17 (previously known as thymus and activation-related chemokine, TARC) and CCL22 (or macrophage-derived chemokine, MDC; chemokine and receptor nomenclature follows Alexander et al. 2011). It has also been reported that CCR4 is a receptor for chemokine-like factor 1, an immune cell chemoattractant which is not a member of the chemokine family (Wang et al. 2006). CCR4 is found on a number of cells of the haematopoietic lineage, for example T cells, platelets (Clemetson et al. 2000), and mast cells (Juremalm et al. 2002). The expression on T cells is restricted to specific subsets as CCR4 has been reported to be expressed on CD25^+^ regulatory T cells (Iellem et al. 2001), skin-homing (cutaneous lymphocyte antigen^+^) T cells (Campbell et al. 1999) and T_H_2 and T_H_17 but not T_H_1 helper T cells (Bonecchi et al. 1998; Lim et al. 2008). The expression of CCR4 on T_H_2 cells has prompted some interest in it as a therapeutic target for asthma and other allergic diseases as the cytokines produced by these cells (interleukins 4, 5, 9, and 13) are thought to induce the pathological changes associated with these diseases (Larche et al. 2003). Indeed, CCR4^+^ T cells have been shown to be elevated at the sites of inflammation in a number of allergic diseases (Panina-Boudignon et al. 2001; Nouri-Aria et al. 2002) and numbers are further increased after allergen challenge (Panina-Boudignon et al. 2001). There are also a number of studies in human disease which have shown that CCL17 and CCL22 are elevated in plasma, serum or at sites of inflammation in patients with a number of allergic or eosinophilic conditions (Lezcano-Meza et al. 2003; Jahnz-Rozyk et al. 2005) and that the levels are correlated with disease severity.

**Figure 1:**
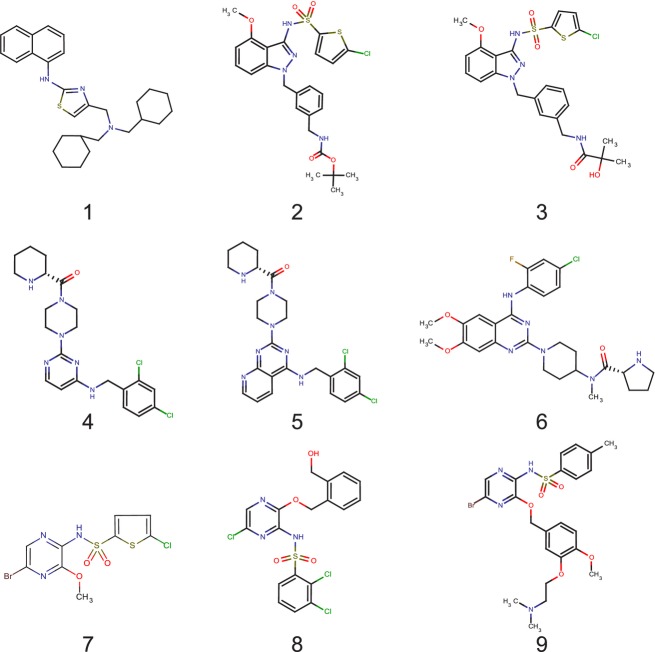
Chemical structures of the low-molecular weight CCR4 antagonists used in this study.

Several classes of low-molecular weight antagonist of CCR4 have now been identified (Purandare and Somerville 2006) and it has recently been reported that at least one of these classes of antagonist may act at an intracellular binding site on CCR4 (Andrews et al. 2008) and must therefore act as allosteric modulators of this receptor. However, in the same study it was clear that the Bristol-Myers Squibb antagonist (compound **5** in Fig. 1) did not bind to this binding site. In this report, we demonstrate that the interactions of a range of CCR4 antagonists (see Fig. 1 and Table 1), are consistent with the presence of two distinct binding sites for low-molecular weight antagonists on CCR4 and that both of these sites are distinct from the binding site for chemokines suggesting that CCR4 has three spatially distinct ligand-binding sites. Some theory required for the interpretation of the antagonist interaction studies is developed in the Appendix.

**Table 1:** The sources of the low-molecular weight antagonists used in this study

Compound	Source patent
1	US7144903B2 (Amgen)
2	WO2010097395A1 (GSK)
3	WO2010097395A1 (GSK)
4	WO2004020584A2 (Bristol-Myers-Squibb)
5	WO2004020584A2 (Bristol-Myers-Squibb)
6	WO2007111227A1 (Astellas)
7	WO2003051870A1 (Astra Zeneca)
8	WO2003059893A1 (Astra Zeneca)
9	US20060004010A1 (Ono)

## Materials and Methods

### Chemokine-stimulated increases in cellular F-actin content

Blood was taken from normal volunteers who had taken no medication within the previous 10 days and chemokine-induced increases in the filamentous (F)-actin content of CD4^+^ CCR4^+^ T cells were measured as previously described (Slack and Hall 2012). Briefly, the peripheral blood mononuclear cells (PBMC) were isolated and stained with fluorescein isothiocyanate-conjugated anti-human CD4 and phycoerythrin-conjugated anti-CCR4 antibodies. The cells were then incubated with antagonist or vehicle (0.1% dimethylsulphoxide [DMSO]) for 30 min at 37°C before stimulation with agonist for 15 sec. The assay was terminated by addition of 3% formaldehyde. The fixed cells were stained with Alexa fluor-647 phalloidin and the mean fluorescence intensity of 1000 CD4^+^ CCR4^+^ cells per sample was determined. This was expressed as a fraction of the mean intensity of the CD4^+^ CCR4^−^ cells in the same sample.

Acquisition of the blood samples was approved by the Hertfordshire Research Ethics Committee and all donors gave informed consent prior to donation.

### Cell culture and membrane preparation

Chinese hamster ovary (CHO)-K1 cells expressing CCR4 under Geneticin selection (CHO-CCR4) were grown in a 95% O_2_/5% CO_2_ atmosphere in Dulbecco's modified eagle medium F12 nutrient mix containing 5% heat-inactivated dialysed foetal bovine serum, 2 mmol·L^−1^ L-gln, and 0.5 mg·mL^−1^ Geneticin. Membranes were prepared from the CHO-CCR4 cells as previously described (Slack and Hall 2012).

### Radioligand binding

#### [^125^I]CCL-17 binding studies

Inhibition of the binding of [^125^I]CCL-17 to CHO-CCR4 membranes was determined using a scintillation proximity assay (SPA) as previously described (Slack and Hall 2012). To allow quantification of the number of binding sites, saturation binding experiments were also performed by filtration. These studies were performed with 20 μg·mL^−1^ membrane protein at room temperature (20–22°C) in SPA-binding buffer (20 mmol·L^−1^ HEPES, 100 mmol·L^−1^ NaCl, 10 mmol·L^−1^ MgCl_2_, 10 μg·mL^−1^ saponin, 0.1% bovine serum albumin (BSA) adjusted to pH 7.4 with KOH) in a total volume of 500 μL. Non-specific binding (NSB) was determined in the presence of 10 nmol·L^−1^ CCL22. Plates were incubated with gentle agitation for 2 h and the reaction terminated by rapid filtration on a Brandel harvester (Brandel Inc. Gaithersburg, MD) through GF/C filter papers presoaked in 0.3% polyethylenimine. Samples were washed three times with ice-cold 0.5 mol·L^−1^ NaCl solution and filters allowed to dry before the amount of bound radioligand was measured using a Packard Cobra II Gamma Counter (PerkinElmer LAS UK Ltd., Beaconsfield, UK). All experiments were performed in the presence of 1% DMSO.

#### [^3^H]antagonist binding studies

[^3^H]antagonist binding experiments were performed as previously described (Slack et al. 2011) with minor modifications. Assays were performed at room temperature (20–22°C) in SPA binding buffer, without BSA, incubation was done for 2 h in a total volume of 1.4 mL. Saturation binding experiments contained 14 μg·mL^−1^ membrane protein, while inhibition experiments were performed at 50 μg·mL^−1^ protein. NSB was determined in the presence of 10 μmol·L^−1^ of the unlabelled compound. Inhibition curves were constructed in the presence of approximately 1 nmol·L^−1^ [^3^H]**5** or approximately 0.7 nmol·L^−1^ [^3^H]**8**. Binding was terminated by filtration on a Brandel harvester through GF/B filter papers presoaked in 0.3% v/v polyethylenimine ([^3^H]**5**) or water ([^3^H]**8**). Filters were washed three times with ice-cold distilled water. The amount of radioligand bound was measured by liquid scintillation spectroscopy, in Ultima-Flo™M (PerkinElmer LAS UK Ltd.), using a TriCarb 2900 TR liquid scintillation counter (PerkinElmer LAS UK Ltd). All experiments were performed in the presence of 1% DMSO.

### Materials

All cell culture media and reagents were purchased from Gibco (Invitrogen Ltd., Paisley, UK). DMSO was obtained from Fisher Scientific UK Ltd. (Loughborough, UK). All other chemicals were purchased from Sigma-Aldrich Co. Ltd. (Gillingham, UK) unless otherwise stated. Chemokines were obtained from R&D systems (Abingdon, UK), Peprotech (London, UK) or ALMAC (Craigavon, UK) (CCL22). [^125^I]CCL-17 (specific activity 2200 Ci mmol^−1^) were obtained from PerkinElmer LAS UK Ltd. [^3^H]**5** and [^3^H]**8** (specific activity 37 and 53 Ci mmol^−1^ respectively) were synthesized by GE Healthcare UK Ltd. (Little Chalfont, UK). Small molecule antagonists were synthesized by Respiratory CEDD Medicinal Chemistry, GlaxoSmithKline.

### Data analysis

Concentration-response curves were fitted with a Hill function of the form

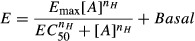
where, [*A*] is the agonist concentration, *E* is the response to that concentration of agonist, *E*_max_ is the maximal response to the agonist, *Basal* is the level of activity in the absence of agonist and *n*_*H*_ is the Hill coefficient.

To quantify the effects of antagonists in the functional assays, concentration-ratios (*DR*) were estimated. In cases where the antagonist caused a change in the maximal response, the *DR* was calculated at the response level corresponding to half the maximal response in the presence of the antagonist (this is justified in the Appendix). When the effect of a combination of antagonists was investigated, the concentration-ratio was calculated at half of the maximal response for the curve with the lowest maximal response of the set (see Appendix).

Binding inhibition curves were fitted with a Hill function of the following form

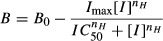
where, [*I*] is the inhibitor concentration, *B* is the level of binding in the presence of that concentration of inhibitor, *I*_max_ is the maximal level of inhibition of binding, *B*_0_ is the level of radioligand binding in the absence of the inhibitor and *n*_*H*_ is the Hill coefficient. Where inhibitors reduced the binding to a level which wasn't significantly different from NSB, the affinity (*K*_*i*_) was determined using the Cheng-Prusoff correction (Cheng and Prusoff 1973; Leff and Dougall 1993). Where specific binding was only partially inhibited and data quality allowed, the interaction was assumed to be allosteric and the data were fitted with the following equation (Ehlert 1988)

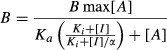
where, *A* is the radioligand, *I* is the inhibitor, *K*_*a*_ is the dissociation constant of the radioligand, *K*_*i*_ is the dissociation constant of the inhibitor and *α* is the binding cooperativity constant.

## Results

**Figure 2:**
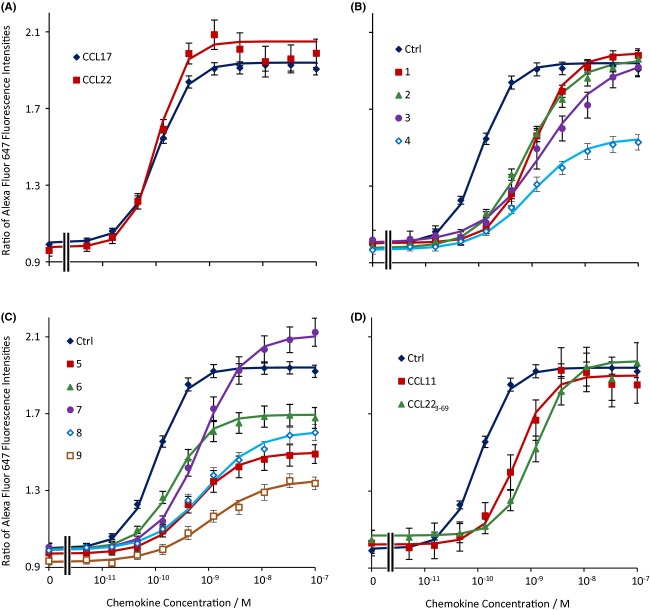
Effects of the antagonists on increases in the F-actin content of human CD4^+^ CCR4^+^ T cells. (A) The effects of CCL22 and CCL17 alone. (B) The effects of CCL17 alone (ctrl) or in the presence of 3 μmol·L^−1^ **1**, 10 μmol·L^−1^ **2**, 1 μmol·L^−1^ **3** or 300 nmol·L^−1^ **4**. (C) The effects of CCL17 alone (ctrl) or in the presence of 30 nmol·L^−1^ **5**, 100 nmol·L^−1^ **6**, 3 μmol·L^−1^ **7**, 300 nmol·L^−1^ **8** or 100 nmol·L^−1^ **9**. (D) The effects of CCL17 alone (ctrl) or in the presence of 1 μmol·L^−1^ CCL11, or 300 nmol·L^−1^ CCL22_3-69_. Data are the mean of the replicate determinations (as specified in Table 2 or the text) and vertical bars show the SEM. Curves show the Hill function generated from the mean of the fit parameters.

CCL17 and CCL22 induced concentration-dependent increases in the F-actin content of human CD4^+^ CCR4^+^ cells. The pEC_50_ of CCL17 was 9.97 ± 0.02 (*n* = 69) and that of CCL22 was 9.99 ± 0.04 (*n* = 17) (Fig. 2A). The effects of the low-molecular weight antagonists on the increase in F-actin content of the T cells induced by CCL17 are summarized in Figure 2B,C, and Table 2. The effects of the antagonistic chemokines are shown in Figure 2D. Compounds **6**, **7**, and **8** caused a small but statistically significant decrease in the F-actin content of the cells (*P* < 0.05, paired *t*-test) while the other low-molecular weight antagonists had no significant effect. CCL11 and CCL22_3-69_ were also without effect on the F-actin content of the cells. With the exception of compound **3**, all of the low molecular weight antagonists significantly changed the maximal response to CCL17 (*P* values are noted in Table 2). Compounds **4**, **5**, **6**, **8**, and **9** were insurmountable while compounds **1**, **2**, and **7** increased the maximal response to this agonist (for contrast, in the remainder of the text this phenomenon will be referred to as suprasurmountability), although the effect of compound **1** was relatively small compared with that of the other two compounds. The antagonistic chemokines had no significant effect on the maximal response to CCL17.

**Table 2:** The effects of the antagonists on CCL17-induced increases in the F-actin content of human CD4^+^ CCR4^+^ T cells when used alone

Treatment (*n*)	Concentration	pEC_50_	Basal	Maximum	Log(slope)	Log(*DR*^1^)	% Inhibition^2^
Control (69)	–	9.97 ± 0.02	1.00 ± 0.01	1.94 ± 0.03	0.17 ± 0.01	–	–
**1** (42)	3 μ mol·L^−1^	8.99 ± 0.04	1.00 ± 0.02	1.99 ± 0.03	0.10 ± 0.01	0.97 ± 0.03	−3.5 ± 1.4*
**2** (32)	10 μ mol·L^−1^	9.11 ± 0.06	0.98 ± 0.02	1.96 ± 0.04	0.00 ± 0.02	0.80 ± 0.04	−8.9 ± 2.8**
**3** (3)	1 μ mol·L^−1^	8.79 ± 0.22	1.00 ± 0.04	1.96 ± 0.07	−0.12 ± 0.05	1.16 ± 0.26	−5.3 ± 10.8
**4** (21)	300 n mol·L^−1^	9.03 ± 0.09	0.97 ± 0.02	1.55 ± 0.04	−0.05 ± 0.02	1.12 ± 0.06	38.4 ± 2.2***
**5** (19)	30 n mol·L^−1^	9.26 ± 0.09	0.97 ± 0.02	1.50 ± 0.05	0.01 ± 0.02	0.96 ± 0.06	42.1 ± 2.3***
**6** (14)	100 n mol·L^−1^	9.65 ± 0.06	1.00 ± 0.02	1.69 ± 0.06	0.10 ± 0.02	0.61 ± 0.05	31.0 ± 1.9***
**7** (15)	3 μ mol·L^−1^	9.14 ± 0.05	0.99 ± 0.02	2.11 ± 0.07	0.03 ± 0.02	0.81 ± 0.03	−15.1 ± 2.7***
**8** (27)	300 n mol·L^−1^	9.07 ± 0.07	0.99 ± 0.02	1.61 ± 0.04	−0.06 ± 0.03	1.19 ± 0.06	40.3 ± 2.0***
**9** (9)	100 n mol·L^−1^	8.98 ± 0.13	0.93 ± 0.02	1.36 ± 0.03	−0.08 ± 0.03	1.23 ± 0.07	49.4 ± 2.4***
CCL11 (7)	1 μ mol·L^−1^	9.29 ± 0.05	1.02 ± 0.07	1.90 ± 0.10	0.17 ± 0.04	0.64 ± 0.07	−2.8 ± 3.7
CCL22_3-69_ (9)	100 n mol·L^−1^	8.92 ± 0.06	1.07 ± 0.04	1.97 ± 0.07	0.15 ± 0.03	0.91 ± 0.07	−2.1 ± 4.9

1 Concentration-ratio: calculated relative to the response at the midpoint of the curve in the presence of the inhibitor.

2 Percentage inhibition of the maximal response to CCL17.

* *P* < 0.02

** *P* < 0.005

*** *P* < 10^−4^ (Student's *t*-test).

**Figure 3:**
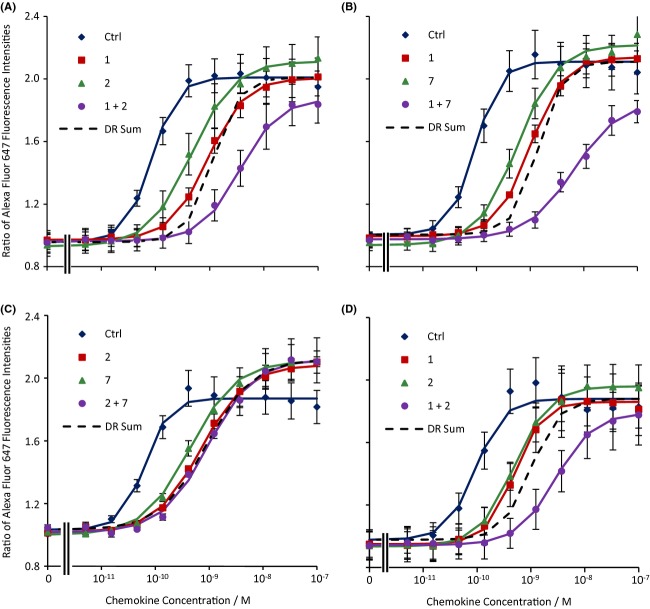
The effects of combinations of antagonists on chemokine-induced increases in the F-actin content of human CD4^+^ CCR4^+^ T cells. (A) The effects of CCL17 alone (ctrl) or in the presence of 3 μmol·L^−1^ **1**, 10 μmol·L^−1^ **2** or **1** and **2** at these concentrations. (B) The effects of CCL17 alone (ctrl) or in the presence of 3 μmol·L^−1^ **1**, 3 μmol·L^−1^ **7** or **1** and **7** at these concentrations. (C) The effects of CCL17 alone (ctrl) or in the presence of 10 μmol·L^−1^ **2**, 3 μmol·L^−1^ **7** or **2** and **7** at these concentrations. (D) The effects of CCL22 alone (ctrl) or in the presence of 3 μmol·L^−1^ **1**, 10 μmol·L^−1^ **2** or **1** and **2** at these concentrations. Data are the mean of the replicate determinations (as specified in Table 3 or the text) and vertical bars show the SEM. Continuous curves shown the Hill function generated from the mean of the fit parameters. The dashed curves show the expected position of a concentration-response curve shifted by the sum of the DRs of the two antagonists.

As an initial approach to determining the minimum number of binding sites available to CCR4 antagonists, we determined the effects of combinations of the antagonists on CCL17-induced increases in CD4^+^ CCR4^+^ cell F-actin content. The effects of coincubation with compounds **1** and **2** are shown in Figure 3A. The DR of the combination (49.0 [22.9, 105], *n =* 4) was much greater than the sum of the DRs of the two antagonists alone (13.7) and close to their product (45.9). A similar pattern of behaviour was observed on coincubation with compounds **1** and **7** (Fig. 3B). However, in this case, the DR of the combination (90.0 [65.5, 124], *n =* 4) was greater than the product of the individual DRs (49.8). The sum was 14.7. Interestingly, coincubation of CCL17 with **2** and **7** (Fig. 3C) resulted in a DR of 10.8 (5.6, 21.0) (*n =* 3), which was similar to the sum of their individual DRs (14.0) and markedly less than their product (46.2).

This suggests that **1** binds to a site distinct from that to which **2** and **7** bind but that **2** and **7** may bind to a common site (see Appendix 1). Hence, we examined the interactions of these compounds with the other antagonists to explore their binding site specificity. The interactions of the other low-molecular weight antagonists with compounds **1** and **2** are summarized in Table 3. Coincubation with compound **1** gave a DR greater than the sum of the individual DRs with compounds **3**, **8**, and **9** while coincubation of these compounds with compound **2** resulted in DRs close to the sum of the individual DRs. The converse was true of compounds **4** and **5** whose effects approximately summated with compound **1** and were greater than additive with those of compound **2**. Interestingly, compound **6** had an approximately additive interaction with both **1** and **2**. Coincubation of CCL22_3-69_ with either **1** or **2** resulted in a DR which was greater than the sum of the DRs for the individual antagonists and this was also true of coincubation of CCL11 with **1**. This is also summarized in Table 3. Coincubation of the cells with CCL22_3-69_ and CCL11 resulted in a DR (12.4 [4.2, 36.3], *n =* 3) which was very close to the sum of the DRs of the individual antagonists (13.9, product 36.2). No formal statistical analysis was performed on the interaction data as the statistical distribution of the sum of two DRs or its logarithm is not known. The effects of the combinations of antagonists on the maximal response to CCL17 are summarized in Table 4. Figures illustrating the effects of coincubation of cells with **1** or **2** and the other antagonists are provided in the Supporting information.

**Table 3:** The effects of combinations of compounds 1 or 2 and the other antagonists on CCL17-induced increases in the F-actin content of human CD4^+^ CCR4^+^ T cells

Compound^1^	DR^2^ ipo**1**	Sum	Product	DR^2^ ipo**2**	Sum	Product
**3** (3, 3)	91.1 (10.5, 790)	18.7	86.2	18.3 (5.4, 62.5)	22.4	113
**4** (4, 5)	19.0 (8.6, 42.0)	19.6	95.8	30.2 (11.4, 79.9)	16.2	57.5
**5** (3, 4)	12.8 (4.2, 38.7)	10.8	28.8	27.8 (15.7, 49.4)	18.4	37.9
**6** (4, 3)	13.5 (8.7, 21)	13.3	37.6	14.0 (5.0, 38.9)	10.0	24.7
**8** (3, 3)	83.7 (20.3, 566)	20.8	108	11.3 (6.4, 19.9)	17.7	73.5
**9** (5, 5)	183.9 (91.2, 372)	24.1	146	21.1 (9.2, 48.6)	21.2	73.1
CCL11: 1 (4)	25.1 (8.5, 73.9)	10.0	24.8	ND	ND	ND
CCL22_3-69_ (4, 5)	67.7 (46.3, 99.0)	17.3	73.6	23.4 (13.2, 41.4)	12.4	37.8

ND, not determined.

1 Concentrations were the same as stated in Table 2. Numbers in parentheses are the number of replicates for the determinations in the presence of **1** then **2**.

2 Values in parentheses after the DRs show the 95% confidence interval.

**Table 4:** The effects of combinations of compounds 1 or 2 and the other antagonists on the maximal increase in the F-actin content of human CD4^+^ CCR4^+^ T cells in response to CCL17

Compound^1^	i.p.o.**1**	Alone^2^	**1** 2	i.p.o.**2**	Alone^2^	**2** 2
**3** (3, 3)	36.5 ± 7.1	−5.3 ± 10.8	−6.2 ± 5.2	3.6 ± 3.1	−5.3 ± 10.8	−8.6 ± 4.7
**4** (4, 5)	17.6 ± 2.1	37.5 ± 13.4	−4.4 ± 3.6	31.1 ± 5.1	36.6 ± 3.6	−0.7 ± 3.5
**5** (3, 4)	22.8 ± 6.5	36.0 ± 3.4	−2.5 ± 1.0	49.6 ± 4.9	54.9 ± 1.5	2.5 ± 2.4
**6** (4, 3)	4.1 ± 2.5	28.9 ± 1.7	−0.1 ± 2.2	−2.4 ± 10.3	33.8 ± 3.2	−29.4 ± 9.9
**7** (4, 3)	19.2 ± 5.5	−16.0 ± 1.6	−4.1 ± 3.5	−34.3 ± 10.6	−33.2 ± 4.2	−29.8 ± 9.7
**8** (3, 3)	49.5 ± 6.1	40.5 ± 1.3	−1.5 ± 3.1	9.7 ± 2.9	42.5 ± 4.2	−17.6 ± 5.0
**9** (5, 5)	68.1 ± 4.1	49.3 ± 3.6	−8.8 ± 1.9	32.9 ± 8.2	49.3 ± 3.6	−0.7 ± 3.5
CCL11: 1 (4)	−8.9 ± 2.8	−1.7 ± 2.5	−3.2 ± 5.2	ND	ND	ND
CCL22_3-69_ (4, 5)	1.7 ± 3.4	7.3 ± 7.1	2.7 ± 4.2	−0.5 ± 3.7	−9.7 ± 5.1	0.9 ± 11.7

ND, not determined.

1 Concentrations were the same as stated in Table 2. Numbers in parentheses are the number of replicates for the determinations in the presence of **1** then **2**.

2 The mean and sem of the percentage inhibition of the maximal response to CCL17 in the presence of the antagonists determined in the same experiments as the effects of their combination.

The effect of the combination of compounds **1** (3 μmol·L^−1^) and **2** (10 μmol·L^−1^) was also determined on CCL22-induced increases in CD4^+^ CCR4^+^ T cells (Fig. 3D). Coincubation with the two antagonists caused a shift in the CCL22 concentration-response curve (DR = 35.5 [28.0, 45.0], *n =* 3) that was much greater than the sum of the individual DRs (10.6). Indeed, in this case it was somewhat larger than the product of the DRs (27.8). In contrast to their effects on CCL17, neither compound alone nor their combination had a significant effect on the response to high concentrations of CCL22. Also, **1**, **4**, **5**, **6**, **7**, and **8** (at the concentrations tested against CCL17) had no effect on increases in the F-actin content of this T-cell population in response to CXCL12, an agonist of CXCR4, in cells from two donors (data not shown).

Binding site interactions were further explored in radioligand binding assays. In saturation binding experiments, [^125^I]CCL17 bound to CHO-CCR4 membranes with affinity 0.15 nmol·L^−1^ (pK_D_ = 9.82 ± 0.06, *n =* 4). The saturating amount of specific binding was 0.73 ± 0.06 pmol per mg membrane protein. [^3^H]**5** bound with affinity 1.4 nmol·L^−1^ (pK_D_ = 8.87 ± 0.06, *n* = 3) and, at saturation, labelled 10.0 ± 2.8 pmol of binding sites per mg protein while [^3^H]**8** bound with affinity 0.28 nmol·L^−1^ (pK_D_ = 9.56 ± 0.08, *n* = 3) and labelled 11.0 ± 1.0 pmol binding sites per mg protein at saturation. The number of binding sites labelled by the two tritiated antagonists was not significantly different (Student's *t*-test). None of the radioligands showed a measurable level of specific binding to membranes from CHO-K1 cells which had not been transfected with CCR4.

**Figure 4:**
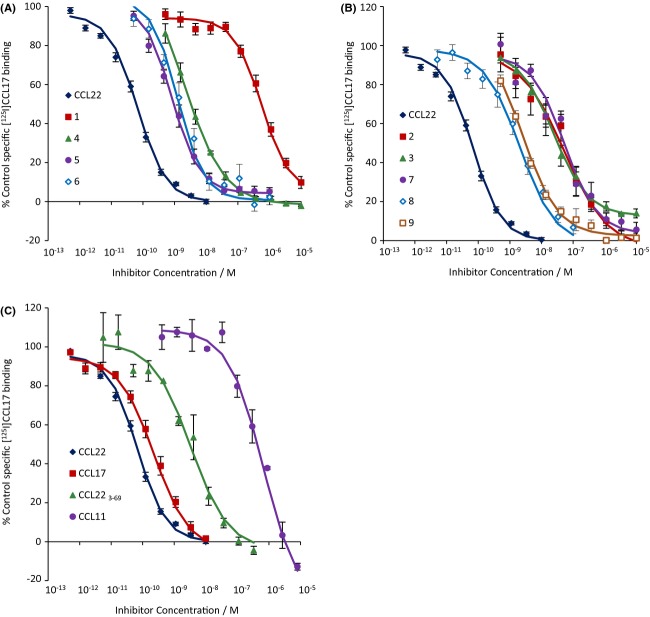
Inhibition of [^125^I]CCL17 binding to membranes from CHO-CCR4 cells by: (A) CCL22, **1**, **4**, **5** or **6**; (B) CCL22, **2**, **3**, **7**, **8** or **9**; (C) CCL22, CCL17, CCL22_3-69_ or CCL11. Data are the mean of at least three separate determinations and vertical bars show the SEM. Curves show the Hill function generated from the mean of the fit parameters.

**Figure 5:**
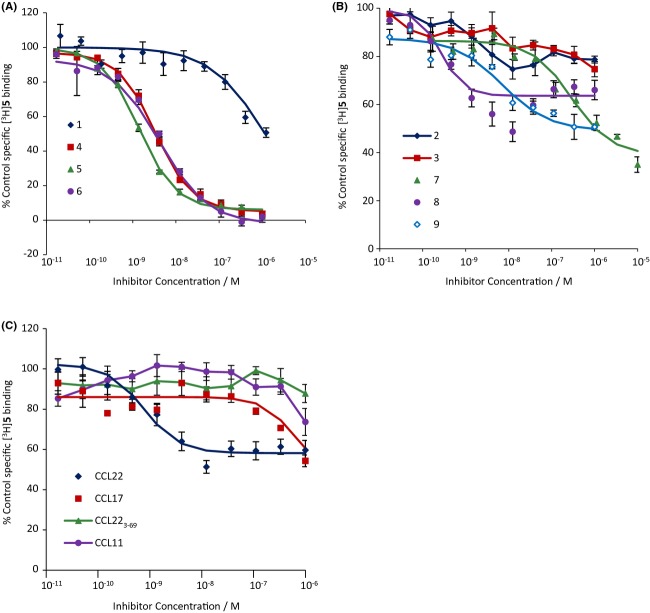
Inhibition of [^3^H]**5** binding to membranes from CHO-CCR4 cells by: (A) **1**, **4**, **5** or **6**; (B) **2**, **3**, **7**, **8** or **9**; (C) CCL22, CCL17, CCL22_3-69_ or CCL11. Data are the mean of three separate determinations and vertical bars show the SEM. Curves show the Hill function generated from the mean of the fit parameters.

**Figure 6:**
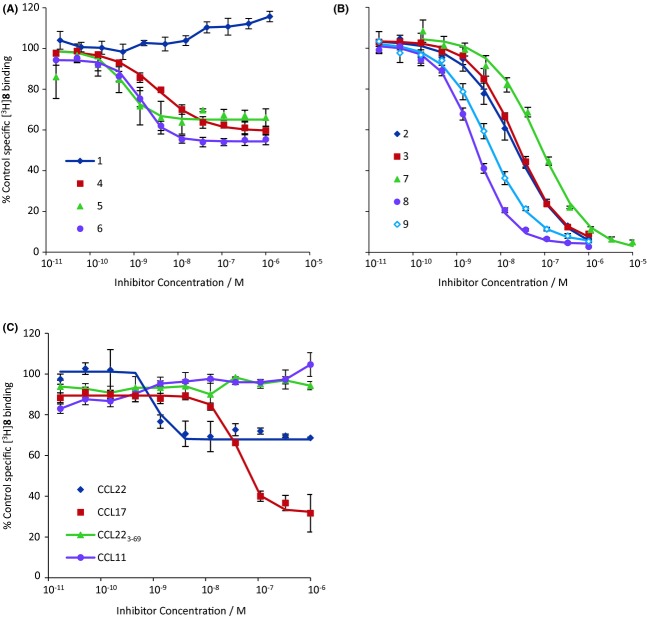
Inhibition of [^3^H]**8** binding to membranes from CHO-CCR4 cells by: (A) **1**, **4**, **5** or **6**; (B) **2**, **3**, **7**, **8** or **9**; (C) CCL22, CCL17, CCL22_3-69_ or CCL11. Data are the mean of three separate determinations and vertical bars show the SEM. Curves show the Hill function generated from the mean of the fit parameters.

The binding of [^125^I]CCL17 was inhibited to a level which was not significantly different from NSB by all of the chemokines and all of the low-molecular weight antagonists except **3**, **8**, and **9** (Fig. 4, summarized in Table 5). These three compounds caused a maximum of 84.9 ± 0.7%, 93.1 ± 1.9%, and 90.5 ± 3.0% inhibition, respectively, and were analysed assuming an allosteric interaction. When effects on the binding of [^3^H]**5** or [^3^H]**8** were determined (Figs. 5A,B and 6A,B, Table 5), the low molecular weight antagonists clearly fell into two groups: those which displaced [^3^H]**5** to its NSB but only partially inhibited [^3^H]**8**, if at all, (**4**, **5**, and **6**) and those which did the converse (**2**, **3**, **7**, **8**, and **9**). Compound **1** was of limited solubility under the conditions of the tritiated antagonist binding assays, precipitating at concentrations above 1 μmol·L^−1^. It was not, therefore, possible to generate complete inhibition curves for this compound. However, lower concentrations of **1** did inhibit the binding of [^3^H]**5** while they did not inhibit the binding of [^3^H]**8** (indeed there may have been an increase in the binding). The chemokines had only limited effects on the binding of either tritiated ligand (Figs. 5C and 6C, Table 5). Indeed, the antagonistic chemokines had no effect at concentrations below 1 μmol·L^−1^.

**Table 5:** The binding affinities and cooperativity factor (α) values for unlabelled antagonists and chemokines against [^125^I]CCL17, [^3^H]compound 5 and [^3^H]compound 8 in CHO CCR4 membranes. Data are the mean ± SEM of at least three separate determinations with 95% confidence limits shown in parentheses where appropriate

Radioligand	[^125^I]CCL17		[^3^H]compound**5**	[^3^H]compound**8**
Compound	pK_i_	α	pK_i_	pK_i_
**1**	6.34 ± 0.07	–	6.21 ± 0.06	<6.00
**2**	7.74 ± 0.41	–	ND	8.26 ± 0.05
**3**	8.21 ± 0.14^1^	0.14 (0.09, 0.21)	ND	8.22 ± 0.01
**4**	8.56 ± 0.08	–	8.76 ± 0.06	ND
**5**	9.10 ± 0.09	–	9.14 ± 0.03	ND
**6**	8.70 ± 0.21	–	8.66 ± 0.05	ND
**7**	7.53 ± 0.05	–	ND	7.56 ± 0.04
**8**	9.04 ± 0.17^1^	0.03 (0.02, 0.04)	ND	9.19 ± 0.04
**9**	8.74 ± 0.09^1^	0.03 (0.02, 0.05)	ND	8.73 ± 0.07
CCL17	9.64 ± 0.10	–	ND	ND
CCL22	10.2 ± 0.05	–	ND	ND
CCL22_3-69_	8.17 ± 0.09	–	ND	ND
CCL11	6.17 ± 0.06	–	ND	ND

ND: Ehlert equation not fitted due to poor definition of the individual inhibition curves.

1 Data derived from fitting the equation of Ehlert (1988).

## Discussion

In this report, we have studied interactions between CCR4 antagonists to investigate the number of binding sites on the receptor. To enable the interpretation of the functional experiments, theory was developed to describe the effects of combinations of allosteric modulators on the response to an agonist (Appendix). The key results of this analysis are that the DRs caused by two allosteric modulators that act at the same site are, generally, maximally additive while those of two modulators acting at distinct sites are likely to be supraadditive. These results are analogous to those describing the interaction of two competitive antagonists (Paton and Rang 1965) and an allosteric and competitive antagonist (Christopoulos and Mitchelson 1994), respectively. However, our treatment considered ligands which are not simply neutral antagonists at the allosteric site and this can modify the behaviour. For example, perfect multiplicativity can only result if at least one of the modulators is surmountable and at most one is an agonist. Furthermore, a surmountable modulator can “protect” the receptors from the effects of an insurmountable compound on the maximal response. This provides another diagnostic criterion for a noncompetitive interaction between modulators as any pair for which this protection is not observed must bind to distinct sites.

In the actin polymerization assays, CCL11 and CCL22_3-69_ behaved as simple surmountable antagonists, consistent with them acting competitively. Of the small molecule antagonists, **4**, **5**, **6**, **8**, and **9** were insurmountable while **1**, **2**, and **7** were suprasurmountable. Insurmountability has two frequent explanations: noncompetitive inhibition or a pseudo-irreversible competitive interaction. Given the rather short agonist contact time in the assay the latter cannot be dismissed outright. Suprasurmountability cannot be the result of a purely competitive interaction. Allosteric ligands can increase the maximal response to an agonist (e.g., Hall (2000)) but this could also be due to an interaction further down the signal transduction cascade. However, neither **1** nor **7** had any effect on the response to CXCL12, an agonist of the related receptor CXCR4, which is also G_i_-coupled (Murphy et al. 2000), suggesting an effect at the level of CCR4.

The small molecule antagonists could be classified into two groups based on their interaction profiles: those which interacted supraadditively with **1** and (sub) additively with **2** and those which did the converse. This suggests that there are two small molecule antagonist binding sites on CCR4, one which binds **1**, **4**, and **5** (site 1) and one which binds **2**, **3**, **7**, **8**, and **9** (site 2). The binding site of **6** is ambiguous as it had an approximately additive interaction with both **1** and **2**. CCL11 and CCL22_3-69_ showed a third profile as their effects were supraadditive with both **1** and **2** but additive with each other indicating an additional binding site for these ligands. Strictly, given the caveats noted in the Appendix, these data are consistent with a *minimum* of three binding sites. Strong negative-binding cooperativity is indistinguishable from competition and hence an additive interaction can also occur when ligands bind to distinct sites.

To test this hypothesis and to further probe the binding site of **6**, radioligand binding experiments were performed with representative small molecule radioligands: [^3^H]**5** for site 1 and [^3^H]**8** for site 2. We also examined effects on [^125^I]CCL17 binding. In CHO-CCR4 cell membranes, all of the ligands inhibited the binding of [^125^I]CCL17 suggesting they do indeed interact with CCR4. Again, the small molecule antagonists divided into two groups: those which inhibited the binding of [^3^H]**5** to NSB but only partially inhibited the binding of [^3^H]**8** (**4** and **6**) and those which did the converse (**2**, **3**, **7**, **8**, and **9**). It was not possible to define a complete inhibition curve for **1** due to its poor solubility under the conditions of the tritiated ligand-binding assays. However, at the concentrations tested, it inhibited the binding of [^3^H]**5** but not that of [^3^H]**8** confirming that **1** binds to site 1. Thus, using two probes which differ from those in the functional studies, the compounds partitioned into the same two sets. This provides further evidence that there are only two binding sites available to bind these compounds as the alternative hypothesis would be quite complex.

CCL11 and CCL22_3-69_ had limited effects on the binding of either tritiated antagonist suggesting that they bind to neither of their binding sites. However, they did inhibit [^125^I]CCL17 binding, suggesting that they bind to CCR4. CCL11 has previously been shown to increase the migration of CCR4-transfected 300-19 cells in response to CCL22 (Sebastiani et al. 2005). This was suggested to result from binding of CCL11 to CCL22 rather than from an interaction via the receptor. However, in our hands CCL11 behaved as a surmountable antagonist of CCL22 and CCL17 in actin polymerization assays (Nalesso et al. 2008; Fig. 2D) and inhibited the binding of [^125^I]CCL17. Although these effects could still be due to an interaction with the chemokines, it is noteworthy that, in Sebastiani et al., CXCL10, and by inference CCL11, did not affect the binding of [^125^I]CCL22. Thus, in our hands, the behaviour of CCL11 is more consistent with that of a simple competitive antagonist. This does not exclude the possibility that CCL11 also binds to CCL17 but this interaction is not apparent under our assay conditions.

One discrepancy noted by Andrews et al. and confirmed in this study was an apparent lack of reciprocity between the effects of the small molecule antagonists on the binding of [^125^I]CCL17 and those of CCL17 on the binding of the small molecules. In Andrews et al., the chemokine had no effect on the binding of the antagonist radioligand. In this report there was a clear inhibitory effect of CCL17 on [^3^H]**8** and of CCL22 on [^3^H]**5** binding, however, in both cases, the inhibition was partial and occurred at concentrations much higher than those required to inhibit the binding of [^125^I]CCL17. This is in contrast to close agreement of the affinities obtained for **5** and **8** in [^125^I]CCL17 and antagonist binding experiments. However, this apparent lack of reciprocity is not inconsistent with the ternary complex model of G-protein activation (De Léan et al. 1980; Appendix). In particular, if an allosteric ligand has no binding cooperativity with the agonist but is an inverse agonist or has negative activation cooperativity, then the ligands only interact when bound to receptor:G-protein (RG) complexes. If RG complexes represent a small proportion of the receptors (and [^125^I]CCL17 labelled approximately 7% as many sites as the tritiated antagonists), the effect of the agonist on labelled antagonist binding may simply not be detectable. If there is binding cooperativity, the midpoint of the agonist inhibition curve will be closer to the affinity for free receptors than that for the RG complex.

As both tritiated antagonists labelled a similar number of binding sites and only bound to membranes from cells transfected with CCR4 both binding sites appear to be present on CCR4. Andrews et al. (2008) showed that **7** and related compounds bound to a site on the intracellular surface of CCR4. Hence, site 2 must correspond to this site. This site binds to aryl sulphonamides and hence to acidic ligands. It is not clear where site 1 resides on the receptor. However, the compounds which bind to this site are all bases. Interestingly, it has been shown that a conserved glutamic acid residue in the seventh transmembrane domain of chemokine receptors (E290 in CCR4) is involved in antagonist binding (Berkhout et al. 2003; Rosenkilde and Schwartz 2006; Wise et al. 2007). It is, therefore, tempting to speculate that site 1 on CCR4 involves this residue. Wherever it is located, it must be an allosteric site given the similarity between the ability of the chemokines to inhibit binding of [^3^H]**5** and [^3^H]**8** and the interaction of CCL22_3-69_ with **1** and **2**. Thus, there appear to be two allosteric sites on CCR4. Hence, the insurmountability of compounds **4**, **5**, **6**, **8**, and **9** is consistent with negative activation cooperativity with CCL17. Indeed, the effects of strictly surmountable allosteric antagonists (i.e., those that only affect orthosteric ligand affinity) cannot become insurmountable when the system does not reach steady state: a finite change in the kinetic constants cannot affect a ligand's ability to saturate the receptors at some finite concentration.

**Figure 7:**
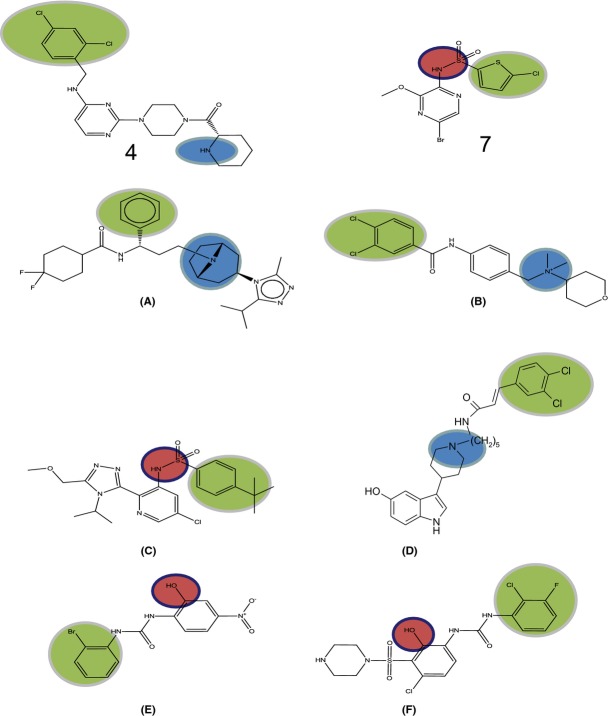
A comparison of selected representative chemokine ligands demonstrating the pharmacophore features of two apparently distinct classes of small molecule. The CCR4 compounds studied here (**4**, **7**) are compared with literature examples of the CCR5 antagonist maraviroc (A), CCR2 antagonists (B–D) and CXCR1–2 compounds (E, F). Lipophilic features are highlighted in green, basic centres in blue, and acidic features in red.

Chemically, the compounds studied here fall into two fundamental classes: the site 1 compounds feature a large lipophilic moiety some distance from a basic centre (as illustrated in Fig. 7); the site 2 compounds are aryl sulphonamides. The former general pharmacophore is well represented among small molecule chemokine antagonists, in particular the CCR5 antagonist maraviroc (A) and a number of CCR2 compounds, for example B (Xia and Sui 2009) and D (Berkhout et al. 2003). Aryl sulphonamides are also found among CCR2 antagonists from Chemocentryx (C) and GlaxoSmithKline (see Xia and Sui 2009) so it is interesting to speculate that CCR2 may have two analogous binding sites.

A characteristic property of the biaryl sulphonamide core is its acidity. It is noteworthy that an acidic biaryl motif has also been described in the putative intracellular antagonists of CXCR1 and CXCR2, for example E (Nicholls et al. 2008) and F (Salchow et al. 2010). Thus, it is further tempting to propose that these general pharmacophores describe the characteristics required of a small molecule to bind to, respectively, the transmembrane and intracellular sites which may be common in several chemokine receptors.

In summary, the use of antagonist interaction and radioligand binding experiments demonstrates that there are three sites on CCR4 at which antagonists can act, the orthosteric site and two allosteric sites.

## Figures and Tables

**Figure A1:**
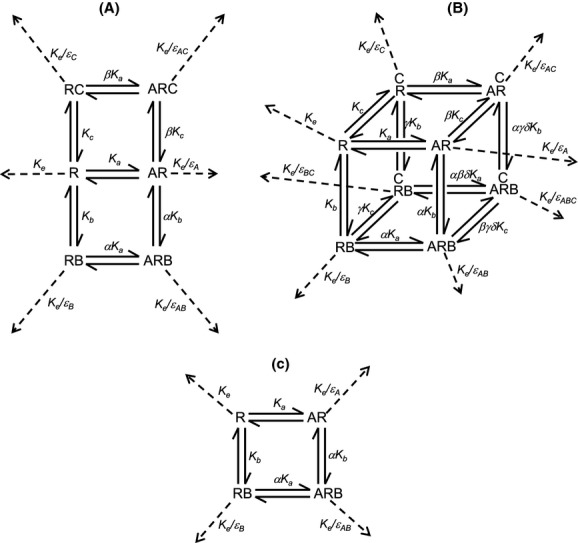
(A) Reaction scheme for a system in which two allosteric modulators (*B* and *C*) compete at a common site to modulate the effects of an orthosteric ligand (*A*). (B) Reaction scheme for a system in which two allosteric modulators bind to distinct sites to modulate the effects of an orthosteric ligand. (C) Reaction scheme for a system in which a single allosteric modulator interacts with an orthosteric ligand.

**Figure A2:**
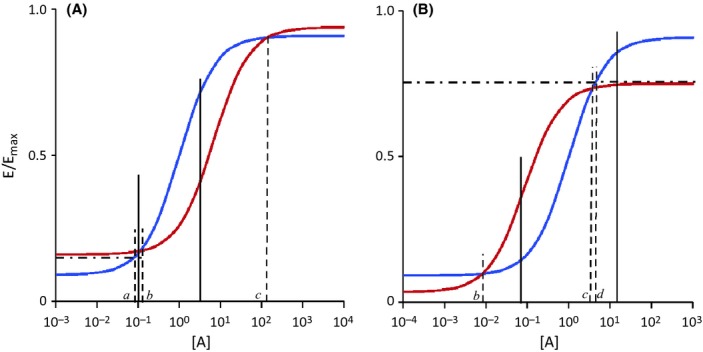
Regions of different DR behaviour according to equation (A2). Concentration-response curves were simulated using equation (A2). In each case [B] = 0 (blue curve) or [B] = 100 (red curve). On each graph *a* marks the concentration of A at which the lower asymptote of the curve in the presence of B intersects the curve in its absence, *b* and *c* mark the concentrations of A at which the curves cross, and *d* marks the concentration of A at which the upper asymptote of the curve in the presence of B intersects the curve in its absence. Note it is only possible to define a meaningful DR when *b* ≪ [A] ≪ *c*. For *a* < [A] < *b* and *c* < [A] < *d* the DR is in the opposite sense and when [A] < *a* or [A] > *d* a DR cannot be defined (and equation (A2) gives a negative value). The vertical black lines mark the upper and lower bounds on the range of [A] derived in Hall (2013) where [A] has little effect on the DR. (A) The modulator is an agonist with positive activation cooperativity and negative binding cooperativity. The parameters for this simulation were: *K*_*a*_ = 10, *K*_*b*_ = 10, *α =* 50, *ε*_*A*_ = 100, *ε*_*B*_ = 2, *ε*_*AB*_ = 400, *χ =* 0.1, *n =* 1. (B) The modulator is an inverse agonist with neutral activation cooperativity and positive-binding cooperativity. The parameters for this simulation were: *K*_*a*_ = 10, *K*_*b*_ = 10, *α =* 0.03, *ε*_*A*_ = 100, *ε*_*B*_ = 0.3, *ε*_*AB*_ = 30, *χ =* 0.1, *n =* 1.

**Figure A3:**
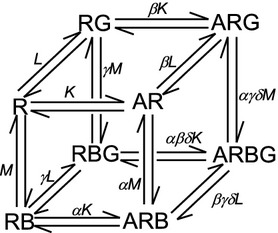
The ternary complex model of G-protein coupled receptor activity with an allosteric modulator. Equilibrium constants are defined in the text.

**Figure A4:**
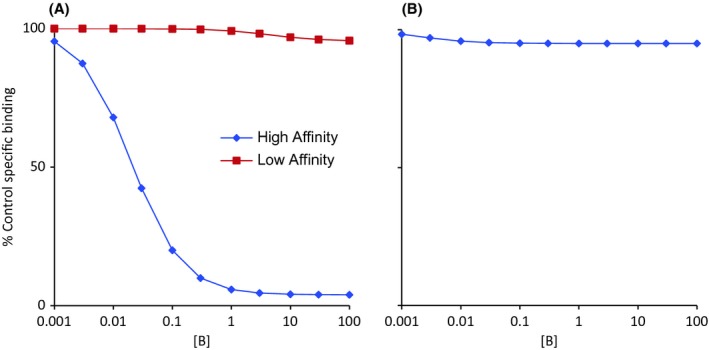
Simulations using equation (A8) of the effects of (A) an inverse agonist on the high-affinity or low-affinity binding of an agonist radioligand or (B) the effect of an agonist on the binding of an inverse agonist radioligand. For (A) the values of the parameters were: [*R*]_T_ = 10, [*G*]_T_ = 0.5, *K =* 1, *L =* 0.001, *M =* 100, *α =* 10^5^, *β =* 0.0001, *δ =* 1. The radioligand was present at its apparent affinity for the high- (0.001) and low-(1) affinity sites, respectively, in this simulation. For (B) the values of the parameters were: [*R*]_T_ = 10, [*G*]_T_ = 0.5, *K =* 100, *L =* 0.001, *M =* 1, *α =* 0.0001, *β =* 10^5^, *δ =* 1. The concentration of the radioligand was 1 in this simulation.

## Appendix

### Effects of combinations of allosteric modulators on responses to an orthosteric agonist

Expressions for the DR of the combination of two competitive antagonists and the combination of a competitive and an allosteric antagonist have previously been derived (Paton and Rang 1965; Christopoulos and Mitchelson 1994). However, to aid interpretation of the data presented in this report, expressions for the effects of the combination of two allosteric modulators are required. In this case, there are two possibilities: competition between the allosteric ligands at the same site and binding of the allosteric ligands to distinct binding sites. The required expressions are derived below using the recently published model of Slack and Hall (2012) as their basis. It should be noted that, as some of the antagonists considered here affect the maximal response to the agonist, this phenomenon has been included in the models. The situations under consideration are shown schematically in Figure A1. For those who prefer not to wade through the mathematical justification, the key results can be stated as follows: the DR of the combination of two allosteric modulators acting at the same site on a receptor is *maximally* the sum of the individual DRs whilewhile that of two modulators acting at different sites should be greater than the sum of the individual DRs. There are some caveats to these statements (particularly when the modulators exhibit negative binding cooperativity with each other) but this provides a useful rule of thumb for interpreting the effects of combinations of allosteric modulators.

#### An operational model of the effects of an allosteric ligand

Prior to considering the effects of combinations of modulators, a novel operational model of the effects of a single allosteric modulator will be derived. This model includes constitutive receptor activity and the possibility that the allosteric modulator is an agonist or inverse agonist in its own right. In the presence of a single allosteric modulator, the systems shown in Figure A1A,B both reduce to that shown in Figure A1C. The properties of this model and its applicability to experimental data have been discussed in detail elsewhere (Hall 2013). However, some complementary details of the derivation and behaviour of the model are presented below to prime the discussion of the models where two allosteric modulators are present. In this scheme, the equilibrium concentration of the free receptor is given by:

where, *K*_*a*_ and *K*_*b*_ are the equilibrium dissociation constants of *A* and *B*, respectively, and *α* is the binding cooperativity constant (Hall 2006). The concentrations of the other receptor species can be obtained from this expression by multiplying [*R*]_*T*_ in the numerator by: [*A*]/*K*_*a*_ for [*AR*]; [*B*]/*K*_*b*_ for [*RB*]; or [*A*][*B*]/*αK*_*a*_*K*_*b*_ for [*ARB*]. The pharmacological stimulus, *S*, is given by *S =* [*R*] + *ε*_*A*_[*AR*] + *ε*_*B*_[*RB*] + *ε*_*AB*_[*ARB*], where the various *ε* factors represent the intrinsic efficacies of the ligands or combinations of ligands indicated by the subscripts (and quantify how effectively they activate the signal transduction pathway relative to the free receptor). Let the response, *E*, be given by

(A1)

where, *χ* = [*R*]_*T*_/*K*_*e*_. In this formulation of the model, the “activation cooperativity” of the ligands is given by *ε*_*AB*_/*ε*_*A*_*ε*_*B*_. Equating the response to equieffective concentrations of the orthosteric ligand in the presence and absence of the allosteric modulator gives the following

Some straight-forward but rather unwieldy algebra then gives the following expression for the *DR* caused by the modulator.

(A2)

The following aspects of this expression are noteworthy. First, as expected, the use of the null method has eliminated the contribution from the signal transduction system – no terms relating to the transducer function (*χ* or *n*) occur in equation (A2). However, the effects of the allosteric modulator do depend on the pharmacological properties of the orthosteric ligand – *K*_*a*_ and *ε*_*A*_ occur in (A2). Indeed, this is also true for the analogous expression for a competing ligand (which can, for example, be derived by letting *α* → ∞ and *ε*_*AB*_ = *ε*_*A*_ in (A2) and is, in fact, its numerator). An important special case of (A2) occurs when *ε*_*B*_ = 1 and *ε*_*AB*_ = *ε*_*A*_ (i.e., when the two ligands only affect each other's binding affinity and the allosteric ligand is a neutral antagonist at the allosteric site). In this case, equation (A2) simplifies to the following

which is (a rearrangement of) the expression derived by Ehlert (1988) with the same assumptions.

The terms in [*A*] in equation (A2) are a consequence of the fact that the concentration-response curves are not parallel when the allosteric ligand has an effect in the absence of *A* or affects its maximal response (as illustrated in Figure A2). When *ε*_*B*_ ≠ 1, the allosteric ligand is an agonist or inverse agonist in its own right. In this case, at low orthosteric ligand concentrations, the term in *K*_*a*_/[*A*] contributes significantly to the DR. There are then concentrations of A for which the concentration-response curve in the presence of a negative allosteric modulator which is an agonist, for example, lie to the left instead of the right of the control curve (the curves cross), resulting in concentration-ratios less than unity in this region. The concentration-ratio can also take negative values. The latter is clearly physically meaningless but, rather than suggesting a flaw in the model, simply reflects the fact that equation (A2) is not mathematically restricted to positive ligand concentrations. Negative concentration-ratios indicate that the one of the curves under comparison can only attain the specified level of response at a negative concentration of *A*. When *ε*_*ΑB*_ ≠ *ε*_*A*_, the allosteric ligand changes the maximal response to *A*. In this case, the term in [*A*]/*K*_*a*_ becomes important when [*A*] is large and causes similar behaviour of the DR (changes in sense and negative values).

The previous paragraph essentially considers the behaviour of the DR near the asymptotes of the concentration-response curves. However, there will often be a range of orthosteric agonist concentrations over which the concentration-response curves in the presence and absence of allosteric ligand are approximately parallel. In this case, the expression for the concentration-ratio simplifies to the following

(see Hall 2013) which shows that, in this range of orthosteric ligand concentrations, a DR can be usefully determined (a brief exploration suggests that a good rule of thumb is to estimate the DR at the half-maximal response level of the most inhibited curve in a set unless this is not possible). Within this range, the concentration of the allosteric modulator which causes a DR of 2 is as follows

and the maximal dose-ratio is

Both of these expressions depend on the intrinsic efficacy of the orthosteric ligand, showing that, even with identical cooperativity constants, there is the potential for a *systematic* difference between orthosteric ligands in the apparent pA_2_ of and maximal shift caused by an allosteric modulator. These expressions do, however, become independent of *ε*_*A*_ when *ε*_*A*_ ≫ *ε*_*B*_ and *ε*_*AB*_ ≈ *ε*_*A*_ > 1, in other words when the modulator is an inverse agonist and/or its effects on the asymptotes of the concentration-response curve to the orthosteric ligand are small. Under these conditions, these expressions simplify to those of an “affinity-only” modulator, that is [*B*]_*DR*=2_ = *αK*_*b*_/(*α* – 2) and *DR*_*max*_ = *α*. In this case, the data can be analysed using the null methods described by Ehlert (1988).

Having established that it is meaningful to define a DR for an allosteric modulator which affects the asymptote(s) of the concentration-response curve to an orthosteric agonist, at least in some circumstances, the effects of combinations of allosteric modulators will be investigated.

#### A model of the effects of two allosteric ligands which bind to the same allosteric site

The binding reaction underlying the model shown in Figure A1A describes the binding of two allosteric modulators to the same site on a receptor in the presence of an orthosteric ligand. In this scheme, the equilibrium concentration of the free receptor is given by:

where, *K*_*a*_, *K*_*b*_, and *K*_*c*_ are the equilibrium dissociation constants of *A*, *B*, and *C*, respectively, and *α* and *β* are the binding cooperativity constants of *A* with *B* and *C* respectively (Hall 2006). The concentrations of the other receptor species can be obtained from this expression by multiplying [*R*]_*T*_ in the numerator by: [*A*]/*K*_*a*_ for [*AR*]; [*B*]/*K*_*b*_ for [*RB*]; [*C*]/*K*_*c*_ for [*RC*]; [*A*][*B*]/*αK*_*a*_*K*_*b*_ for [*ARB*]; or [*A*][*C*]/*βK*_*a*_*K*_*c*_ for [*ARC*]. By analogy with the one allosteric ligand case, the pharmacological stimulus, *S*, can be defined as:

Again, let the response be given by

(A3)

where *χ =* [*R*]_*T*_/*K*_*e*_. Note that when [A] = 0, equation (A3) simplifies to the following

which is simply the equation describing the functional effects of two ligands competing at the same binding site (as expected). Also, when [A] ≫ *K*_*a*_, that is the orthosteric ligand is close to saturating the receptor, equation (A3) simplifies to the following

(A4)

If one of the allosteric ligands is present at a much higher concentration than that of the other, for example let *ε*_*AB*_[*B*]/*αK*_*b*_ ≫ *ε*_*AC*_[*C*]/*βK*_*c*_, then this simplifies to the following

showing that the effect of one allosteric ligand on the maximal response to *A* can dominate that of the other at a sufficiently high concentration. This is consistent with our expectation of competing ligands.

The DR in the presence of two allosteric ligands which bind to the same site on the receptor can be determined in a similar way to that described in Section 1a:

Equating responses to equieffective concentrations of orthosteric ligand in the presence and absence of the modulators and rearranging gives equation (A5).

(A5)

This has a similar linear rational structure to A2 with additive contributions from the two ligands. It is also consistent with the expectation that the effects of one modulator can overwhelm those of another with which it competes if a sufficiently high concentration is used. For ranges of orthosteric ligand concentration where the concentration-response curves in the absence and presence of the allosteric ligands are approximately parallel, this simplifies to the following

Equation (A5) then allows the derivation of the key result of this section for the interpretation of modulator combination experiments which is that, except under certain conditions noted below, *DR*_*BC*_ ≤ *DR*_*B*_ + *DR*_*C*_ (where the subscript indicates the ligand(s) whose DR is being quantified). That is, the DRs of two allosteric ligands acting at the same site are maximally additive. To see this, note that if each term is positive,

As this inequality is also true of any common multiple of both sides, and hence of each other term in equation (A5) (compared to an appropriate form of equation A2), *DR*_*BC*_ ≤ *DR*_*B*_ + *DR*_*C*_ follows:

It is only possible for the DRs to be supraadditive (i.e., for DR_*BC*_ to be greater than *DR*_*B*_ + *DR*_*C*_) if either or both of the variable terms in the denominator of equation (A5) is negative, while leaving the denominator itself positive. Assuming that *ε*_*A*_ > 1 (i.e., *A* is an agonist), this supraadditivity could only be true for all concentrations of *A* if *ε*_*AB*_ ≤ 1 or *ε*_*AC*_ ≤ 1, that is, if *A* is no longer an agonist when *B* or *C* is bound to the receptor. If 1 < *ε*_*AB*_ < *ε*_*A*_, then *DR*_*BC*_ > *DR*_*B*_ + *DR*_*C*_ when [*A*] > *K*_*a*_(*ε*_*AB*_ – 1)/(*ε*_*A*_ – *ε*_*AB*_) (and similarly for *C*). That is if either allosteric ligand converts *A* into a partial agonist, the concentration-ratios become supraadditive above some concentration of *A*. Thus, supraadditivity of concentration-ratios is possible but only if one or both of the allosteric modulators reduce the maximal response to *A*. The effects of coincubation are approximately additive when the denominator of A5 is approximately unity, that is when the terms in [*B*] and [*C*] in the denominator are both much less than unity or the effect of one cancels the effect of the other. In the former case, the behaviour is equivalent to that of the combination of two competitive antagonists.

#### A model of the effects of two allosteric ligands which bind to distinct allosteric sites

Now consider the case of two allosteric modulators that bind to distinct binding sites on the receptor. The reaction scheme for this is shown in Figure A1B (again, see Hall 2006). The equilibrium concentration of the free receptor is given by the following:

where, *K*_*a*_, *K*_*b*_, and *K*_*c*_ are the equilibrium dissociation constants of *A*, *B*, and *C*, respectively, and *α* and *β* are the binding cooperativity constants of *A* with *B* and *C*, respectively, *γ* is the binding cooperativity of *B* with *C* and *δ* is the higher order cooperativity constant for the binding of one of the ligands when the other two are bound to the receptor. The concentrations of the other receptor species can be obtained from this expression by multiplying [*R*]_*T*_ in the numerator by: [*A*]/*K*_*a*_ for [*AR*]; [*B*]/*K*_*b*_ for [*BR*]; [*C*]/*K*_*c*_ for [*CR*]; [*A*][*B*]/*αK*_*a*_*K*_*b*_ for [*ARB*]; [*A*][*C*]/*βK*_*a*_*K*_*c*_ for [*ARC*]; [*B*][*C*]/*γK*_*b*_*K*_*c*_ for [*RBC*]; or [*A*][*B*][*C*]/*αβγδK*_*a*_*K*_*b*_*K*_*c*_ for [*ARBC*]. In this case we define the pharmacological stimulus as *S =* [*R*] + *ε*_*A*_[*AR*] + *ε*_*B*_[*BR*] + *ε*_*C*_[*CR*] + *ε*_*AB*_[*ARB*] + *ε*_*AC*_[*ARC*] + *ε*_*BC*_[*RBC*] + *ε*_*ABC*_[*ARBC*], and proceed as previously,

(A6)

When [*A*] = 0, the response is given by the following

which is, of course, identical to equation (A1) as the system then reduces to that of two ligands interacting allosterically. When [*A*] ≫ *K*_*a*_, the response is as follows

In this case, the terms in [B][C] prevent the effects of high concentrations of one allosteric ligand on the maximal response to the orthosteric ligand from necessarily overwhelming the effects of the other.

Equating the response to equieffective concentrations of *A* in the presence and absence of both *B* and *C* gives the following

(A7)

When the terms in [*A*] can be neglected equation (A7) simplifies to

Equation (A7) contains terms in [*B*][*C*] and hence the effects of one modulator cannot overwhelm those of the other unless these “cross terms” are rendered insignificant (e.g., due to strong negative cooperativity between *B* and *C*). Indeed, the structure of A7 indicates that, for at least some combinations of the parameters, the numerator and denominator will factorize, that is that the interaction is inherently multiplicative. This is further justified below and some special cases are derived, in particular, justifying the choice of a surmountable allosteric modulator as a “standard” for interaction studies. However, as for any two real numbers *w* and *z*, say, which are greater than 2, *wz* > *w* + *z*, this implies that the DRs of allosteric modulators that act at different sites will be supraadditive unless they exhibit (strong) negative-binding cooperativity. This provides the required contrast between the behaviour of modulators acting at the same site (in general DRs maximally additive) and those acting at different sites (likely to be supraadditive).

Returning to the structure of equation (A7), the numerator and denominator will factorize if the coefficients of [*B*][*C*] are the products of the coefficients of [*B*] and [*C*] {(1 + *ax*)(1 + *by*) = (1 + *ax* + *by* + *abxy*)}. Requiring this of the numerator gives the following

which provides the following set of simultaneous equations (by “equating coefficients”).

(*c*) gives *ε*_*B*_ = 1 or *ε*_*C*_ = 1 (*γ =* 0 is excluded as physically meaningless). Letting *ε*_*B*_ = 1, (*a*) gives  and hence  and (*b*) gives  from which . Thus,

which then gives *ε*_*BC*_ = *ε*_*C*_. Similarly, if we assume *ε*_*C*_ = 1, then we obtain *γ =* 1 and *ε*_*BC*_ = *ε*_*B*_. Requiring the same of the denominator gives the following

This gives the following set of simultaneous equations

(*f*) gives *ε*_*AB*_ = *ε*_*A*_ or *ε*_*AC*_ = *ε*_*A*_. Letting *ε*_*AB*_ = *ε*_*A*_, (*d*) gives  and hence  and (*e*) gives . Hence,

from which *ε*_*ABC*_ = *ε*_*AC*_. Similarly, if *ε*_*AC*_ = *ε*_*A*_, then *γδ* = 1 and *ε*_*ABC*_ = *ε*_*AB*_.

The following conditions for the numerator and denominator to factorize have been derived: *γ = δ =* 1, *ε*_*B*_ = 1 and *ε*_*BC*_ = *ε*_*C*_ and/or *ε*_*C*_ = 1 and *ε*_*BC*_ = *ε*_*B*_ and *ε*_*AB*_ = *ε*_*A*_ and *ε*_*ABC*_ = *ε*_*AC*_ and/or *ε*_*AC*_ = *ε*_*A*_ and *ε*_*ABC*_ = *ε*_*AB*_. For example, let *γ = δ =* 1, *ε*_*B*_ = 1, *ε*_*BC*_ = *ε*_*C*_, *ε*_*AC*_ = *ε*_*A*_ and *ε*_*ABC*_ = *ε*_*AB*_, then A7 becomes

Restating this pharmacologically, the interaction between two allosteric modulators which bind to distinct binding sites is perfectly multiplicative if: the modulators exhibit no binding or activation cooperativity with each other, there is no higher order cooperativity, at least one of the modulators is a neutral antagonist and at most one of them exhibits activation cooperativity with the orthosteric ligand. Negative and positive cooperativity between the modulators will result in sub- and supramultiplicative interactions respectively.

#### Comparison of the effects on the maximal response to the orthosteric ligand

It can also be instructive to consider the effects of the combination of allosteric modulators on the maximal response to the orthosteric ligand, particularly when at least one of the orthosteric ligands, *B* say, is surmountable. In this case, the maximal response of a curve in the presence of two competing allosteric modulators must lie between that of the control curve and that of the curve in the presence of the insurmountable (or suprasurmountable) modulator alone (the mathematical details are shown below). If the allosteric ligands bind to different sites this need not be the case, thus should the maximal effect in the presence of the combination fall outside this range, the modulators must bind to distinct sites. Also, it is important to note that the converse is not true: it cannot be assumed that an interaction between modulators is competitive if the maximal response in their combined presence lies between those in their presence individually.

Let the two modulators compete for the same site and assume that *B* is surmountable then, *ε*_*AB*_ = *ε*_*A*_ (to a reasonable approximation) and the expression for the maximal response to A (equation A4) becomes

It is necessary to define a condition under which the maximal response in the presence of *B* and *C* is less than that in the presence of *C* alone, that is

That is *C* must be suprasurmountable. Thus, when *C* is insurmountable the maximal response to *A* in the presence of both allosteric ligands must be greater than that in the presence of *C* alone. Indeed, whether *C* is insurmountable or suprasurmountable, if a surmountable modulator competes with it then the maximal response in the presence of the combination will lie between those in the presence of the modulators individually.

### Apparent nonreciprocal effects of allosteric modulators on agonist binding in the ternary complex model

As Andrews et al. (2008) showed previously and we describe in this report, low-molecular weight CCR4 antagonists can inhibit agonist binding while CCL22 and CCL17 do not inhibit the binding of the antagonists. This appears to violate the principle of microscopic reversibility (Weber 1975) which requires that the effect of the allosteric ligand or orthosteric ligand on the other's affinity is the same (which then allows the unambiguous definition of a thermodynamic constant {the cooperativity constant} which quantifies the interaction). However, as shown below, this behaviour is in fact consistent with the predictions of the ternary complex model of G-protein activation for an allosteric interaction. The model under consideration is shown in Figure A3. The expression for the binding isotherm for this model when neither receptor nor G-protein is in excess was derived in Hall (2006). It is only in this form that the model exhibits both high- and low-affinity binding for agonists and inverse agonists. It is then instructive to consider ligands which exhibit no binding cooperativity (i.e., *γ* = 1). If we denote the radioligand by *A* and the inhibitor by *B*, then the binding isotherm under this assumption is given by equation (A8).

(A8)

where,

*R* is the receptor, *G* is the G-protein, *K* and *M* are the association equilibrium constants of ligands *A* and *B*, respectively, *α* and *β* are their respective intrinsic activities, *δ* is their activation cooperativity and *L* is the affinity of free *R* for *G*.

In this case, the two ligands can only influence each other's binding through their effects on the binding of the G-protein. This is most clearly demonstrated if we consider the forms of equation (A8) when [*G*]_*T*_ = 0 and when [*G*]_*T*_ → ∞. In the former case, [G] = 0 and

(A9)

In the latter case, [G] ≈ [G]_*T*_ and

(A10)

Thus, the allosteric ligand only affects the binding of the orthosteric ligand (and vice versa) to RG complexes and has no effect on the binding to free R. A little care is required in the interpretation of A10 as it suggests that binding to RG would not be influenced by a ligand with no activation cooperativity (*δ =* 1). However, this is an “artefact” of allowing [*G*]_*T*_ to become arbitrarily large and hence saturate the receptor irrespective of the binding affinity. This is not the case for finite [*G*]_*T*_. An agonist radioligand has high affinity for the RG complex and labels it selectively, if it has high efficacy. An inverse agonist allosteric ligand (*β* ≪ 1) or one with strong negative activation cooperativity (*δ* ≪ 1) can then affect the binding of the radioligand. However, an inverse agonist radioligand has high affinity for free R and will label this form of the receptor selectively. An allosteric agonist would then only inhibit the binding of the radioligand from the small proportion of RG complexes to which it binds. Thus, the ternary complex model actually predicts that there can be apparent nonreciprocal effects in binding assays if the effects of agonists and inverse agonists are compared using radioligands with different pharmacological properties. The above behaviours are illustrated in Figure A4.

## Abbreviations

CCL: CC-chemokine ligand
CCR: CC-chemokine receptor
CXCL: CXC-chemokine ligand
CXCR: CXC-chemokine receptor
DMSO: dimethylsulphoxide
DR: concentration ratio
F-actin: filamentous actin
NSB: non-specific binding
PBMC: peripheral blood mononuclear cells
SPA: scintillation proximity assay

## Supporting Information

**Figure S1.** Effects of combinations of CCR4 antagonists on increases in the F-actin content of human CD4+ CCR4+ T cells in response to CCL17.
