# Supplementary material for: Antagonism of human CC-chemokine receptor 4 can be achieved through three distinct binding sites on the receptor
Source: Pharmacol Res Perspect. 2013 Dec 30;1(2):e00019. doi: 10.1002/prp2.19 (PMC4186434; doi:10.1002/prp2.19)
Supplement: Supplementary file 1 — Figure S1. Effects of combinations of CCR4 antagonists on increases in the F-actin content of human CD4+ CCR4+ T cells in response to CCL17. [file prp20001-e00019-SD1.pdf]

Rob J. Slack, Linda J. Russell, Nick P. Barton, [1]Cathryn Weston, [2]Giovanna Nalesso, Sally-Anne Thompson, Morven Allen, Yu Hua Chen, Ashley Barnes, [3]Simon T. Hodgson, David A. Hall.

Antagonism of human CC-chemokine receptor 4 can be achieved through three distinct binding sites on the receptor.

Supplementary figure. Effects of combinations of CCR4 antagonists on increases in the F-actin content of human $CD4^+$ $CCR4^+$ T cells in response to CCL17. The legend to each graph shows the antagonists (ctrl = control). Values are the mean of the replicate determinations (the number of replicates is specified in table 3 of the published article) and vertical bars show the s.e.mean.

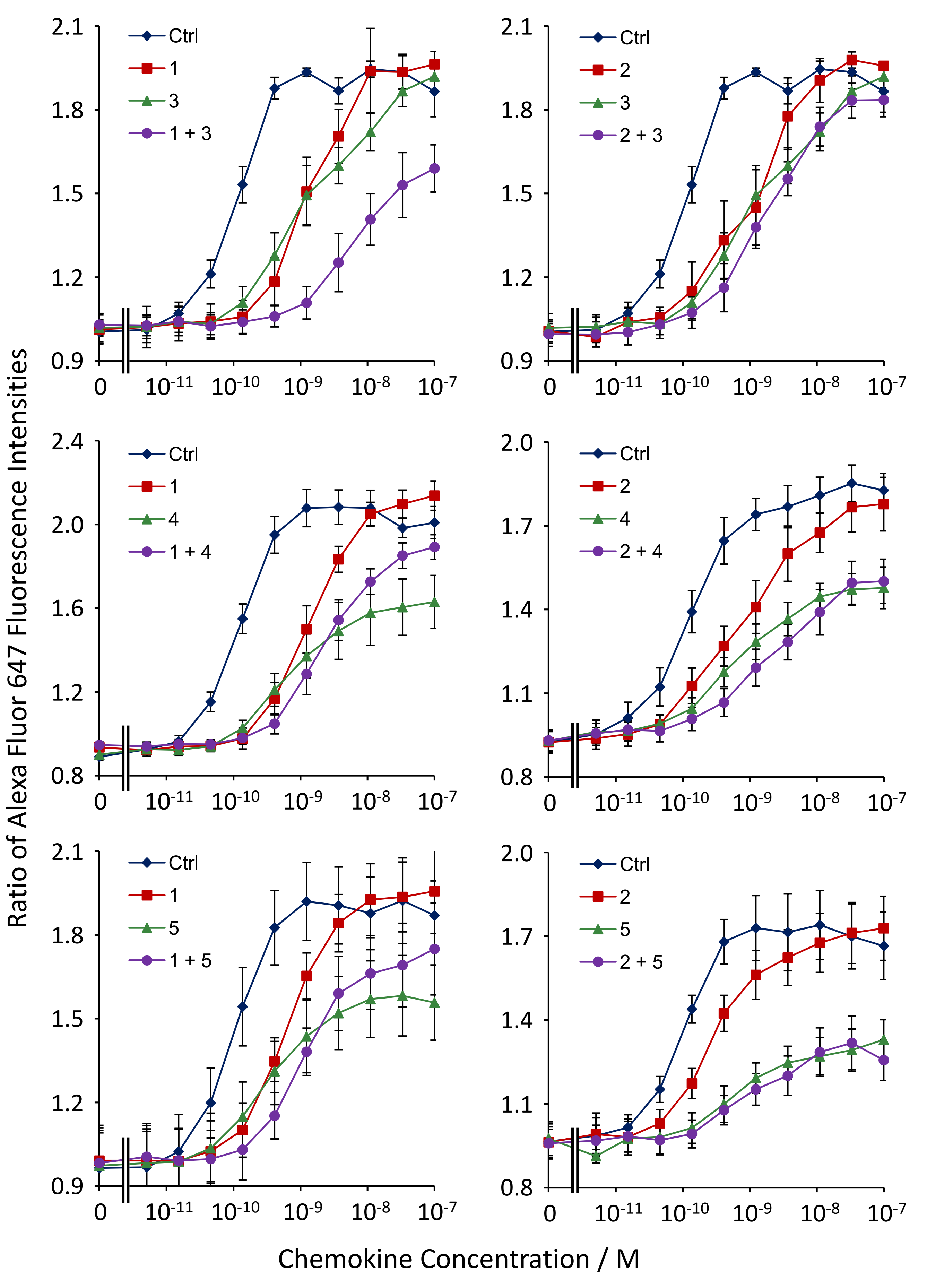

Ratio of Alexa Fluor 647 Fluorescence Intensities
Chemokine Concentration / M
Ctrl
1
3
1 + 3
2
2 + 3
4
1 + 4
2 + 4
5
1 + 5
2 + 5
0
10^{-11}
10^{-10}
10^{-9}
10^{-8}
10^{-7}

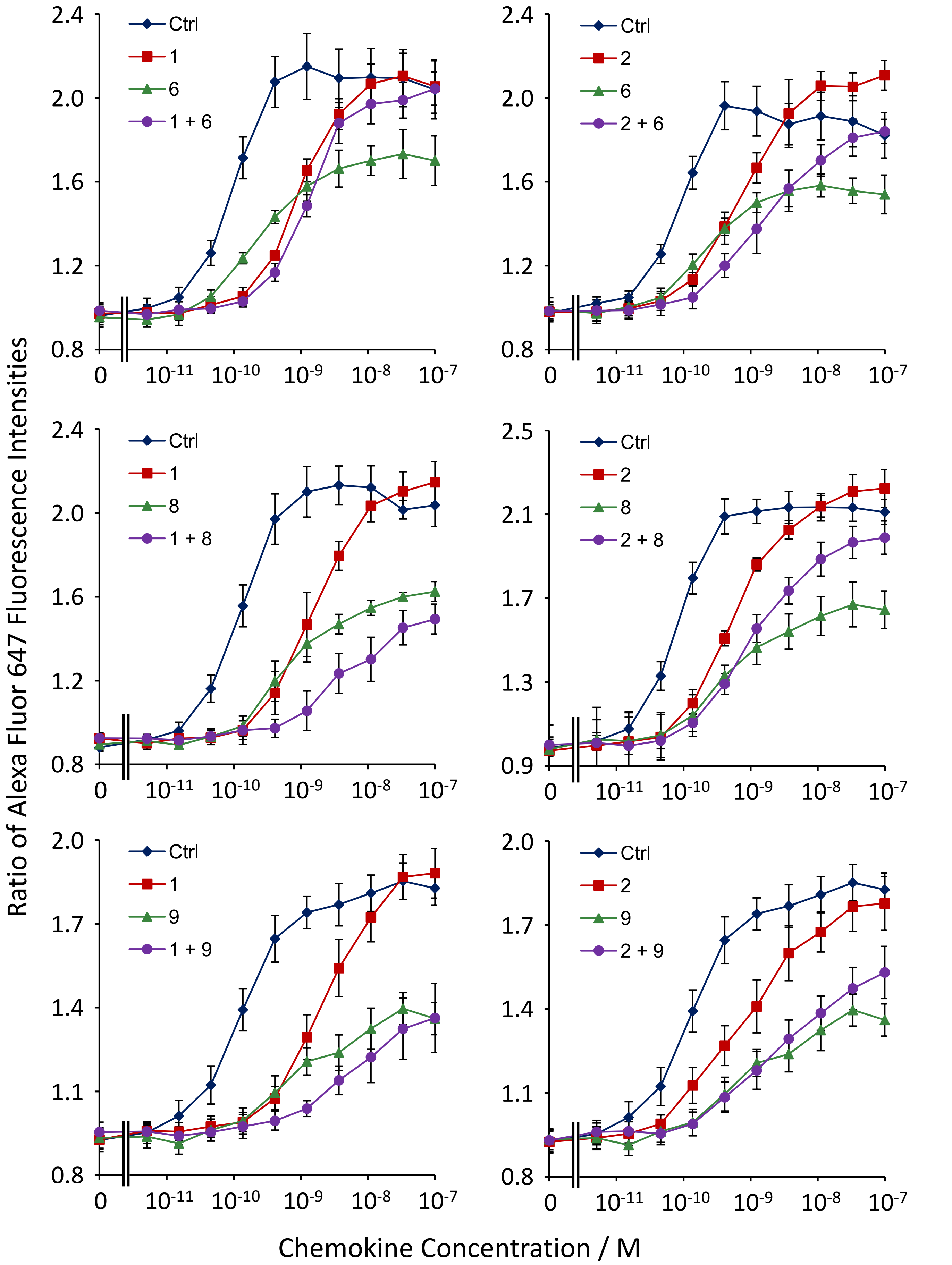

Ratio of Alexa Fluor 647 Fluorescence Intensities
Chemokine Concentration / M
Ctrl
1
6
1 + 6
Ctrl
2
6
2 + 6
Ctrl
1
8
1 + 8
Ctrl
2
8
2 + 8
Ctrl
1
9
1 + 9
Ctrl
2
9
2 + 9
0
10^{-11}
10^{-10}
10^{-9}
10^{-8}
10^{-7}
